# Irisin reduces senile osteoporosis by inducing osteocyte mitophagy through Ampk activation

**DOI:** 10.1016/j.isci.2024.111042

**Published:** 2024-09-26

**Authors:** Honghan Li, Deqing Luo, Wei Xie, Wenbin Ye, Jinlong Chen, Paolo Alberton, Mingzhu Zhang, Eryou Feng, Denitsa Docheva, Dasheng Lin

**Affiliations:** 1 Department of Orthopaedic Surgery, Zhangzhou Affiliated Hospital of Fujian Medical University, Zhangzhou, P.R. China; 2 Department of Orthopaedics, the 909th Hospital, School of Medicine, Xiamen University, Zhangzhou, P.R. China; 3 Experimental Surgery and Regenerative Medicine, Clinic for General, Trauma and Reconstructive Surgery, Ludwig-Maximilians-University (LMU), Munich, Germany; 4 Center of Foot and Ankle Surgery, Beijing Tongren Hospital, Capital Medical University, Beijing, P.R. China; 5 Department of Orthopedic Surgery, Fujian Medical University Union Hospital, Fuzhou, P.R. China; 6 Department of Musculoskeletal Tissue Regeneration Orthopaedic Hospital König-Ludwig-Haus & University of Wuerzburg, Wuerzburg, Germany

**Keywords:** Biological sciences, Physiology, Molecular biology

## Abstract

Irisin, an exercise-induced myokine, is known to be able to regulate bone metabolism. However, the underlying mechanisms regarding the effects of irisin on senile osteoporosis have not been fully elucidated. Here, we demonstrated that irisin can inhibit bone mass loss and bone microarchitecture alteration in senile osteoporosis mouse model. In addition, irisin has effects on bone remodeling that is in favor of bone formation. Remarkably, irisin induced autophagy in osteocytes demonstrated by increased LC3-positive osteocytes, and increased autophagy-related genes and proteins. *In vitro* analysis revealed that Irisin can prevent mitochondrial oxidative damage. Furthermore, irisin can obviously induce osteocyte mitophagy and increased phosphorylation of Ampk and Ulk1. Inhibition of Ampk signaling recapitulated the biological effect of irisin loss, accompanied by the markedly lower expression of Ulk1. Taken together, our findings show that irisin reduces age-related bone loss by inducing osteocyte mitophagy via Ampk-dependent activation of Ulk1.

## Highlights

• Irisin inhibits age-related bone mass loss and bone microarchitecture alteration
• Irisin activates autophagy in the osteocytes
• Irisin induces osteocyte mitophagy
• Irisin increases phosphorylation of Ampk in the MLO-Y4 cells

## Graphical abstract

**Figure:**
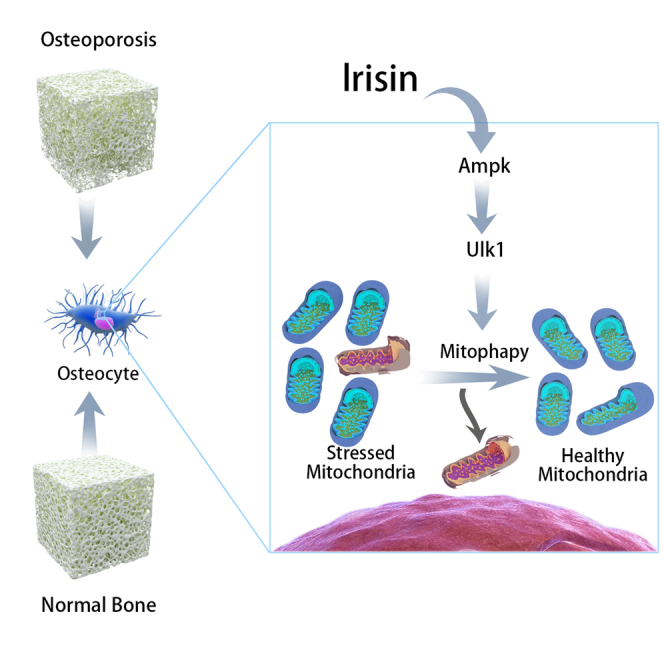

## Introduction

Osteoporosis is a common disease characterized by a systemic impairment of bone mass and microarchitecture that results in fragility fractures. With an aging population, the medical and socioeconomic effect of osteoporosis will increase further.^1^ In the past decades, the pathogenesis of osteoporosis has been linked to tissue, cellular, and molecular processes. Master signals that integrate various endocrine, neuroendocrine, inflammatory, and mechanical stimuli have been defined.^1^ A simple yet effective way to enhance bone mass and architecture is through mechanical stimulation of the resident bone cell population. Exercise improves bone mass and strength that is recommended as baseline treatment in each person with osteoporosis.^1^^,^^2^ Irisin, an exercise-induced myokine, which is secreted by muscle, increases with exercise, and mediates certain favorable effects of physical activity, which could be therapeutic for human metabolic disease and other disorders that are improved with exercise.^3^^,^^4^ Interestingly, recent studies have demonstrated that irisin boosts the bone mass,^5^^,^^6^ and voluntary exercise increases irisin production in bone tissues.^7^ Importantly, the osteocytes are direct targets of irisin.^8^ Osteocytes arise from mature osteoblasts, are imbedded in the cortical matrix, and comprise nearly 90% of the cellular composition of bone tissues.^9^ The osteocyte is seen as the cell at the center and initiator of the bone remodeling process.^10^^,^^11^^,^^12^ There is a marked decline in osteocyte number accompanied by an increase in osteocyte apoptosis with age. In addition, Rochefort et al.^13^ have demonstrated that apoptotic osteocytes have the potential to recruit osteoclasts to the vicinity and result in increasing bone resorption. Therefore, preservation of osteocyte viability might be a potential approach for the treatment of osteoporosis.^14^

Autophagy is an essential metabolic pathway for cell survival, which involved in the degradation of damaged or aging intracellular proteins and to maintain cell homeostasis.^15^ Recently, autophagy has been shown to be an important protective mechanism in osteocyte and impaired autophagy with age play a critical role in the development of senile osteoporosis. Improving the autophagy of osteocyte is one of the promising therapeutic approaches for osteoporosis.^16^^,^^17^ Cumulatively, the previous theoretical framework and research evidence reveal that irisin may be considered a favorable option to induce autophagy, and therefore play an important regulatory role in bone metabolism. Specifically, Kim et al.^8^ demonstrated that identification of irisin receptors as integrins in osteocytes and thermogenic fat suggests that αV family integrin complexes are likely to be the major irisin receptors. However, the results of previous studies on irisin were based on the ovariectomy mouse model or young mice.^6^^,^^8^ The effect of irisin on the senile osteoporosis and the possible mechanisms are not entirely known. Therefore, the purpose of this study was to investigate whether irisin acts to inhibit age-related bone loss through regulating osteocyte autophagy, and determine the possible mechanism underlying in the process that irisin participates in the prevention of senile osteoporosis.

## Results

### Irisin inhibits age-related bone mass loss and bone microarchitecture alteration

**Figure 1:**
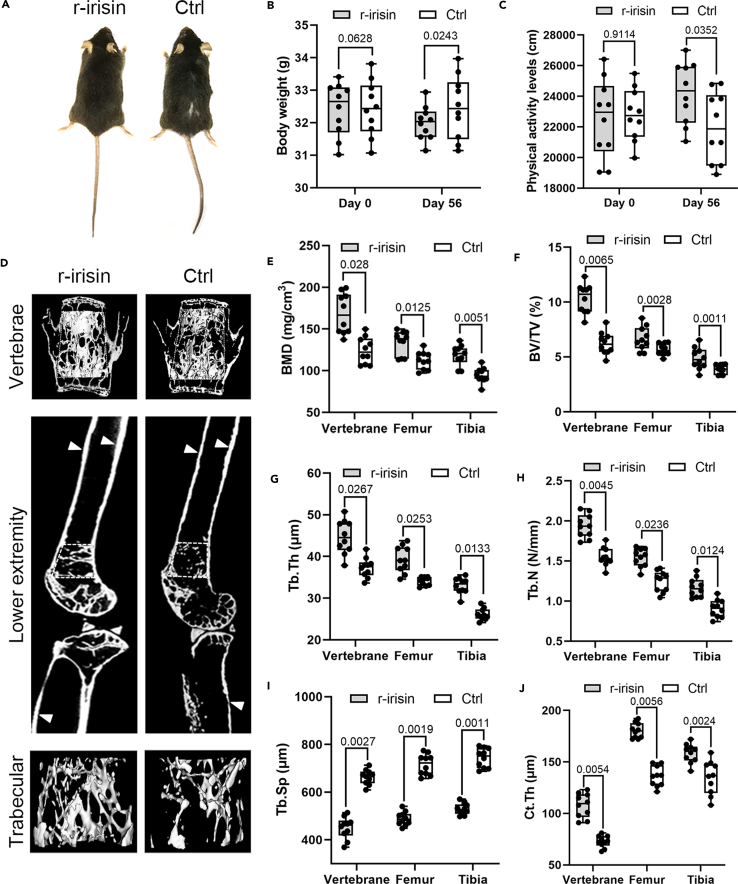
Irisin reduces body weight, enhances physical activity, and inhibits bone mass loss and bone microarchitecture alteration in senile osteoporosis mice (A) Photograph of 24-month-old mice to demonstrate hair loss from control group, whereas r-irisin treatment group had no overt phenotype. (B and C) The body weight was reduced, and the physical activity was enhanced in the treatment group compared to the control group. (D) Three-dimensional images of the L4 vertebrae and the lower extremity. (E–J) Quantitative Micro-CT analysis of BMD, BV/TV, Tb.Th, Tb.N, Tb.Sp, and Ct.Th in the L4 vertebrae, femur, and tibia from two groups mice. All quantitative data were assessed by two-tailed unpaired Student’s t test; *n* = 10 animals. White arrows, cortical bones. Data are represented as mean ± SD.

To investigate the effects of irisin on the senile osteoporosis, we first evaluated the general appearance, body weight, and physical activity in the senile osteoporosis mouse model, which was injected with r-irisin 10 μg kg^−1^ or vehicle per day for 8 weeks. We observed that hair loss was not noticeable (Figure 1A), the body weight was reduced (Figure 1B), and the physical activity was enhanced (Figure 1C) in the r-irisin treatment group when compared with the control group. Micro-CT was performed to detect the bone mass and bone microarchitecture alteration of the L4 lumbar vertebrae, femur, and tibia. Our observation is in line with previous studies indicating a marked effect on trabecular and cortical bone in the treatment group compared to the control group, which was deteriorative microstructure existed (Figure 1D). The bone mineral density (BMD) of L4 lumbar vertebrae, femur, and tibia in the treatment group were higher than the control group (Figure 1E). Trabecular bones microstructure was also determined, and the results showed that the treatment group had higher bone volume over total volume (BV/TV), trabecular thickness (Tb.Th), trabecular number (Tb.N), and lower trabecular separation (Tb.Sp) when compared with the control group (Figures 1F–1I). In addition, Micro-CT analysis also showed that the treatment group had higher cortical thickness (Ct.Th) compared with the control group (Figure 1J). Taken together, our results demonstrated that irisin can reduce body weight, enhance physical activity, and inhibit age-related bone mass loss and bone microarchitecture alteration in the senile osteoporosis mouse model.

### Irisin treatment has effects on bone remodeling that is in favor of bone formation

**Figure 2:**
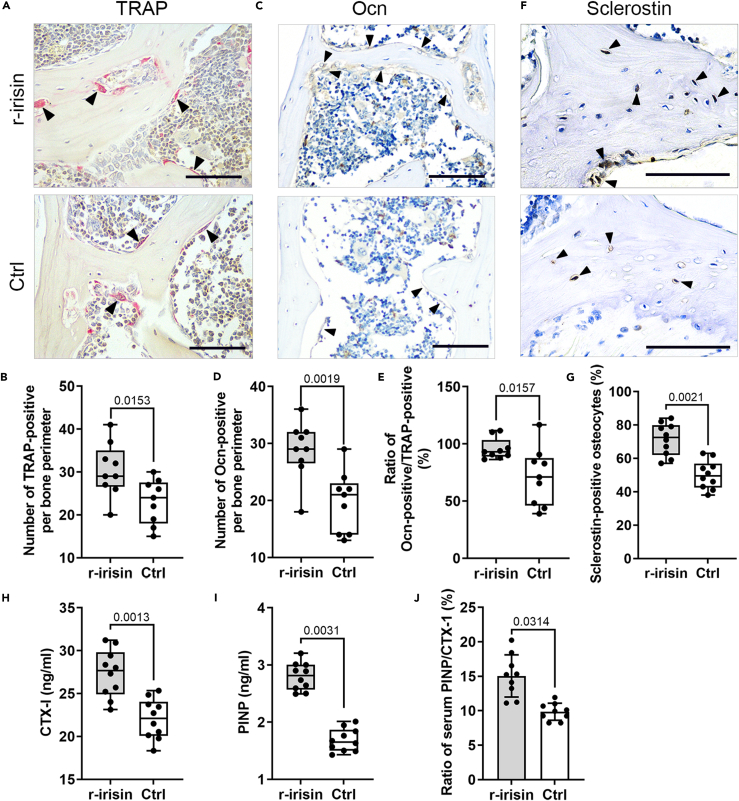
Irisin increases the ratio of osteoblasts/osteoclasts, the protein expression of sclerostin, and the ratio of serum PINP/CTX-I (A and B) TRAP staining and quantification for the L4 lumbar vertebrae bone sections demonstrate increased osteoclast number in the treatment group compared to the control group. (C and D) IHC of the L4 lumbar vertebraes shows the protein expression level of Ocn was increased in the treatment group compared to the control group. (E) Quantification of bone turnover reveals the ratio of Osteocalcin-positive cells to Trap-positive cells was upregulated. (F and G) IHC of the tibias demonstrates increased the protein expression level of sclerostin in the treatment group compared to the control group. (H–J) The level of the CTX-I, PINP, and the ratio of serum PINP/CTX-I were increased in the treatment group when compared with the control group. All quantitative histomorphometry data were assessed by two-tailed non-parametric Mann-Whitney test; *n* = 10 animals. Black arrows, positive cells. Scale bar, 20 μm. Data are represented as mean ± SD.

In a manner consistent with our observations of a decrease in age-related bone loss via irisin treatment, we then evaluated the direct effects of irisin on the bone resorption of osteoclasts and the bone formation of osteoblasts in the two groups. The immunohistochemistry (IHC) staining showed increased numbers of TRAP-positive osteoclasts and the protein expression level of Ocn, a special marker of mature osteoblasts,^18^ in the r-irisin treatment group compared to the control group (Figures 2A–2D). Interestingly, this action was accompanied by dramatically increased osteoclastic bone resorption and osteoblastic bone formation, upregulated ratio of Osteocalcin-positive cells to Trap-positive cells, that inhibited the bone mass loss (Figure 2E). Importantly, irisin also induced the protein expression level of sclerostin, which is produced almost exclusively by osteocytes,^2^^,^^9^ the “command and control” cells of the bone-remodeling unit (Figures 2F and 2G). To further verify the effects of irisin on bone turnover, serum CTX-I and PINP levels in blood to be the reference markers of bone turnover were detected.^19^ Interestingly, the level of the serum CTX-I and PINP were increased in the treatment group when compared with the control group (Figures 2H and 2I). Furthermore, the ratio of serum PINP/CTX-I was significantly increased in the treatment group (Figure 2J).

In addition, we also investigated whether irisin regulates the balance between osteogenic and adipogenic differentiation in hBMSCs, which may be recruited to bone remodeling by irisin. However, our results showed that irisin did not accelerate their commitment toward the osteogenic differentiation (Figures S1A and S1B) or inhibited the adipogenic differentiation (Figures S2A and S2B). Furthermore, qPCR showed that the expression of critical osteogenic or adipogenic regulatory genes such as *RUNX2* and *SP7* (Figures S1C and S1D), or *PPARγ*, *LPL*, and *FABP4* (Figures S2C–S2E) were also similar between the stimulated and the control group. Thus, these results suggest that the irisin treatment leads to increase of bone turnover that is in favor of bone formation in the senile osteoporosis mouse model.

### Irisin activates autophagy in the osteocytes

**Figure 3:**
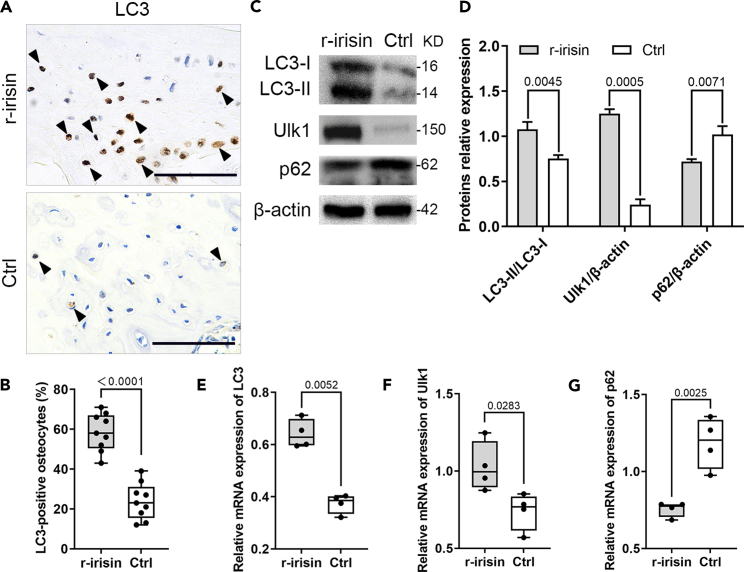
Irisin preserves osteocyte viability through inducing osteocyte autophagy (A and B) Significant upregulation of LC3 protein expression was detected in the femurs using IHC imaging and quantitative analysis in the treatment group compared to the control group (two-tailed non-parametric Mann-Whitney test; *n* = 10 animals). (C and D) Western blotting confirmed that LC3-I, LC3-II, Ulk1 protein expression levels were higher, and p62 was lower in the treatment group (two-tailed non-parametric Mann-Whitney test; *n* = 10 animals). (E–G) Significant upregulation of autophagy-related genes *LC3* and *Ulk1*, and downregulation of p62 were detected in treatment group by qPCR analysis (two-tailed unpaired Student’s t test; *n* = 10 animals). Black arrows, positive cells. Scale bar, 20 μm. Data are represented as mean ± SD.

Irisin is known to be regulated by exercise. It is generally believed that autophagy plays an important role in remodeling of skeletal muscle in response to exercise.^20^^,^^21^^,^^22^^,^^23^ Meantime, recent studies have shown that autophagy also plays a critical role for the maintenance of bone homeostasis.^17^^,^^24^^,^^25^^,^^26^ For these reasons, we then explored whether irisin has protective effects on the senile osteoporosis through inducing autophagy. IHC staining results demonstrated that treatment with r-irisin significantly increased the number of the microtubule-associated protein, LC3, a marker for autophagosome assembly, positive osteocytes in osteocyte-enriched lumbar vertebras (Figures 3A and 3B). Western blotting analysis confirmed that the protein expression of LC3-I, LC3-II, and Ulk1, a downstream regulator of autophagy/mitophagy, were significantly higher in the treatment group compared with the control group (Figures 3C and 3D). Moreover, irisin also stimulated the ratio of LC3-II to LC3-I. Consistently, qPCR showed that *LC3* and *Ulk1* mRNA expression level were significantly higher in the treatment group compared to the control group (Figures 3E and 3F). In addition, the protein and mRNA expression level of p62/SQSTM1, a selective substrate for autophagy and scaffold in autophagosomes,^27^ decreased significantly under irisin treatment, indicating enhanced autophagy in the treatment process (Figures 3C, 3D, and 3G). Taken together, our results demonstrated that irisin can preserve osteocyte viability through inducing osteocyte autophagy, thereby regulating bone remolding in the senile osteoporosis mouse model.

### Irisin prevents mitochondrial oxidative damage in the MLO-Y4 cells

**Figure 4:**
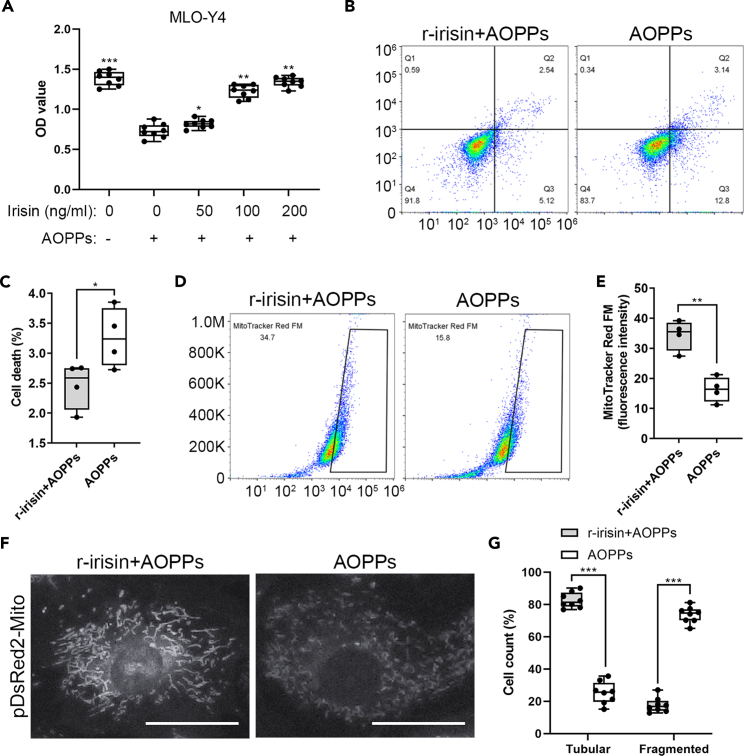
Irisin exhibits increased proliferation potential and mitochondrial activity, and reduced cell death in the MLO-Y4 cells (A) MLO-Y4 cells were treated with the indicated concentrations of irisin and 200 μg/mL AOPPs, followed by analysis of cell viability using MTT (two-tailed unpaired Student’s t test; *n* = 3 independent experiments). (B–E) MLO-Y4 cells were treated with 100 ng/mL irisin and 200 μg/mL AOPPs, followed by analysis of cell death and mitochondrial activity using flow cytometry (two-tailed unpaired Student’s t test; *n* = 3 independent experiments). (F and G) MLO-Y4 cells were transiently transfected with the mitochondrial marker pDsRed2-Mito and treated with 100 ng/mL irisin and 200 μg/mL AOPPs for quantitative analysis of the mitochondrial morphology. The number of cells containing tubular and fragmented mitochondria was expressed as percentage of total counted cells (two-tailed non-parametric Mann-Whitney test; *n* = 3 independent experiments). ^∗^*p* < 0.05; ^∗∗^*p* < 0.01; ^∗∗∗^*p* < 0.001. Scale bar, 20 μm. Data are represented as mean ± SD.

Our findings suggest that irisin activates autophagy in the osteocytes. To further understand the mechanisms underlying the role of irisin on the senile osteoporosis, we then evaluated the direct effects of irisin on osteocytes *in vitro*. In our study, using the oxidative state that the medium was added 200 μg/mL advanced oxidation protein products (AOPPs) to characterize osteoporosis microenvironment,^28^^,^^29^^,^^30^^,^^31^ the murine osteocyte-like cell line MLO-Y4 cells were treated with 50, 100, or 200 ng/mL r-irisin, respectively. 3-(4,5-Dimethyl-2-thiazolyl)-2,5-diphenyl-2-H-tetrazolium bromide (MTT) assay showed an increase in cell viability in the irisin treatment group, which increased in the irisin dose-dependent manner (Figure 4A). Flow cytometry analysis demonstrated that the MLO-Y4 cells under the oxidative stress with 100 ng/mL r-irisin significantly decreased the percentage of cell death compared to the control group treated with vehicle (Figures 4B and 4C). Importantly, at this state, the mitochondrial activity of the MLO-Y4 cells was also significantly induced in the irisin treatment group when compared with the control group using MitoTracker Red FM analysis (Figures 4D, 4E, S3A, and S3B), which is in line with previous report.^32^ Furthermore, in order to define the role of irisin in mitochondrial physiology, we performed pDsRed2-Mito plasmid analysis in the presence and absence of irisin for the MLO-Y4 cells under the oxidative stress. Our results demonstrated the presence of a significant amount of tubular mitochondrial in the irisin treatment group compared with the fragmented ones observed in the control group (Figures 4F and 4G). Collectively, these data demonstrated that irisin exhibits increased proliferation potential and mitochondrial activity, and reduced cell death in the MLO-Y4 cells.

### Irisin induces osteocyte mitophagy

**Figure 5:**
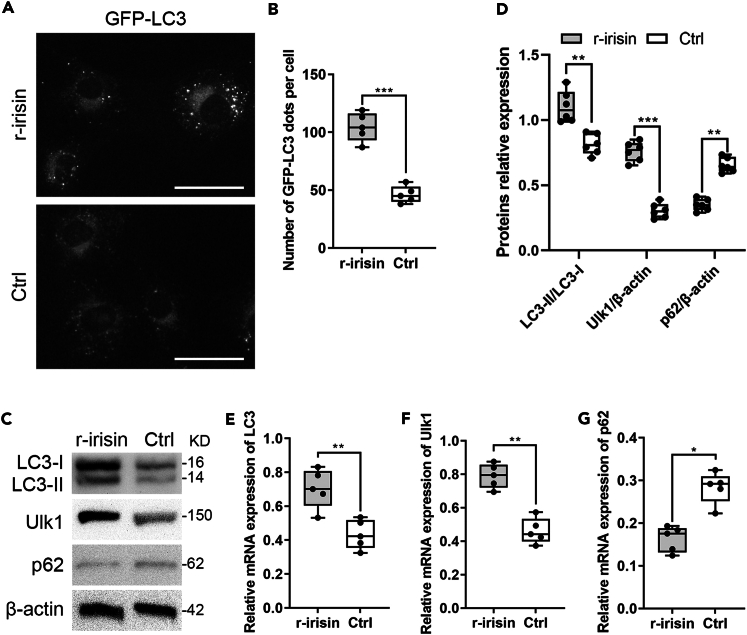
Irisin obviously induces osteocyte mitophagy and increases LC3 turnover (A and B) MLO-Y4 cells transiently expressing GFP-LC3 were treated with 100 ng/mL irisin and 200 μg/mL AOPPs and the average number of GFP-LC3 dots per cell was quantified (two-tailed non-parametric Mann-Whitney test; *n* = 3 independent experiments). (C and D) Western blotting confirmed that LC3-I, LC3-II, Ulk1 protein expression levels were higher, and p62 was lower in the treatment group compared to the control group (two-tailed non-parametric Mann-Whitney test; *n* = 3 independent experiments). (E–G) Significant upregulation of autophagy-related genes *LC3* and *Ulk1*, and downregulation of p62 were detected in treatment group by qPCR analysis (two-tailed unpaired Student’s t test; *n* = 3 independent experiments). ^∗^*p* < 0.05; ^∗∗^*p* < 0.01; ^∗∗∗^*p* < 0.001. Scale bar, 20 μm. Data are represented as mean ± SD.

Since irisin activates autophagy in the osteocytes and exhibits increased mitochondrial activity, we further characterize the role of irisin in mitochondrial signaling and development of mitochondrial network. Overall, mitophagy is essential mechanisms regulating mitochondrial network-related signaling, mitochondrial oxidative stress and mitochondria-associated cell death events.^33^^,^^34^^,^^35^^,^^36^^,^^37^^,^^38^ In our study, the MLO-Y4 cells were suffered from oxidative stress with AOPPs, only a small number of GFP-LC3 dots, a marker of autophagosomes, were detected. The number of these GFP-LC3 dots increased with irisin treatment (Figures 5A and 5B). Western blotting analysis exhibited that the protein expression levels of LC3-I, LC3-II, and Ulk1 were significantly higher in the treatment group compared with the control group. Specifically, irisin also stimulated the ratio of LC3-II to LC3-I (Figures 5C and 5D). In addition, qPCR showed that *LC3* and *Ulk1* mRNA expression level were significantly higher in the treatment group (Figures 5E and 5F). However, the protein and mRNA expression level of p62/SQSTM1 decreased significantly under irisin treatment, indicating enhanced the autophagy in the treatment process (Figures 5C, 5D, and 5G). Altogether, these findings provide the first direct evidence of irisin induced osteocyte mitophagy associated with increased mitochondrial oxidative stress. We also provide *in vitro* evidence for an early upregulation of LC3-II and Ulk1, both of which may play key roles in stimulating osteocyte mitophagy.

### Irisin increases phosphorylation of Ampk in the MLO-Y4 cells

**Figure 6:**
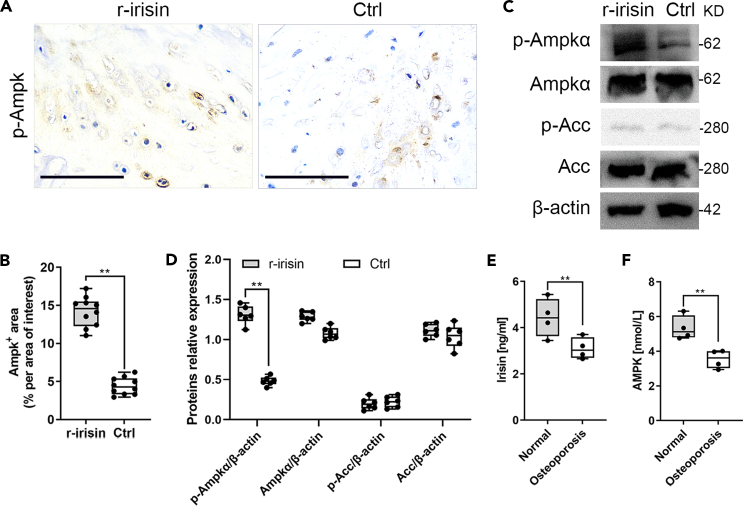
Irisin increases the protein expression levels of *p*-Ampk in bone tissue (A and B) Significant upregulation of *p*-Ampk protein expression was detected in the femurs using IHC imaging and quantitative analysis in the treatment group compared to the control group (two-tailed non-parametric Mann-Whitney test; *n* = 10 animals). (C and D) Western blotting confirmed that *p*-Ampkα protein expression was higher, and no noticeable change in Ampkα, *p*-Acc, and Acc in the treatment group compared to the control group (two-tailed non-parametric Mann-Whitney test; *n* = 10 animals). (E and F) Decreased immunoreactivity due to age-related-decreased irisin and AMPK expression were detected in the osteoporosis patients by using target-specific ELISA (two-tailed unpaired Student’s t test; *n* = 10–12 samples). ^∗∗^*p* < 0.01. Scale bar, 20 μm. Data are represented as mean ± SD.

Autophagy is promoted by Ampk, which is a key energy senor and regulates cellular metabolism to maintain energy homeostasis.^39^^,^^40^ Meantime, Ampk activation plays an important role in the hormonal regulation of bone remodeling.^41^^,^^42^ Ampk activation increases bone formation while deletion of *Ampkα1* leads to decreased bone mass. Recent studies have revealed that one ancestral function of Ampk is to promote mitochondrial health, and multiple newly discovered targets of Ampk are involved in various aspects of mitochondrial homeostasis, including mitophagy.^43^^,^^44^^,^^45^ Therefore, we next determined whether irisin plays an important regulatory role in the osteocyte mitophagy that targets Ampk directly.^46^ In our study, IHC staining with *p*-Ampk antibody indicated induced Ampk activity in the bone tissues from r-irisin-injected mice compared with vehicle-injected mice (Figures 6A and 6B). In agreement with irisin-activating bone formation signaling mechanisms, we also found that irisin increased *p*-Ampkα protein in the treatment group than in the control group, but no noticeable change the protein expression level of Ampkα, and its downstream substrate Acc and *p*-Acc in the bone tissues using western blotting analysis (Figures 6C and 6D). Irisin levels in circulation have been shown to be lower than normal in patients with osteoporosis.^47^^,^^48^^,^^49^ In our study, we also explored whether irisin and AMPK were produced at senile osteoporosis less than normal people. As expected, decreased immunoreactivity due to age-related-decreased irisin and AMPK expression were detected in the osteoporosis patients by using target-specific ELISA (Figures 6E and 6F). Taken together, irisin plays an important regulatory role in osteocyte mitophagy that targets *p*-Ampkα directly.

### Inhibition of Ampk signaling recapitulates the biological effect of irisin loss

**Figure 7:**
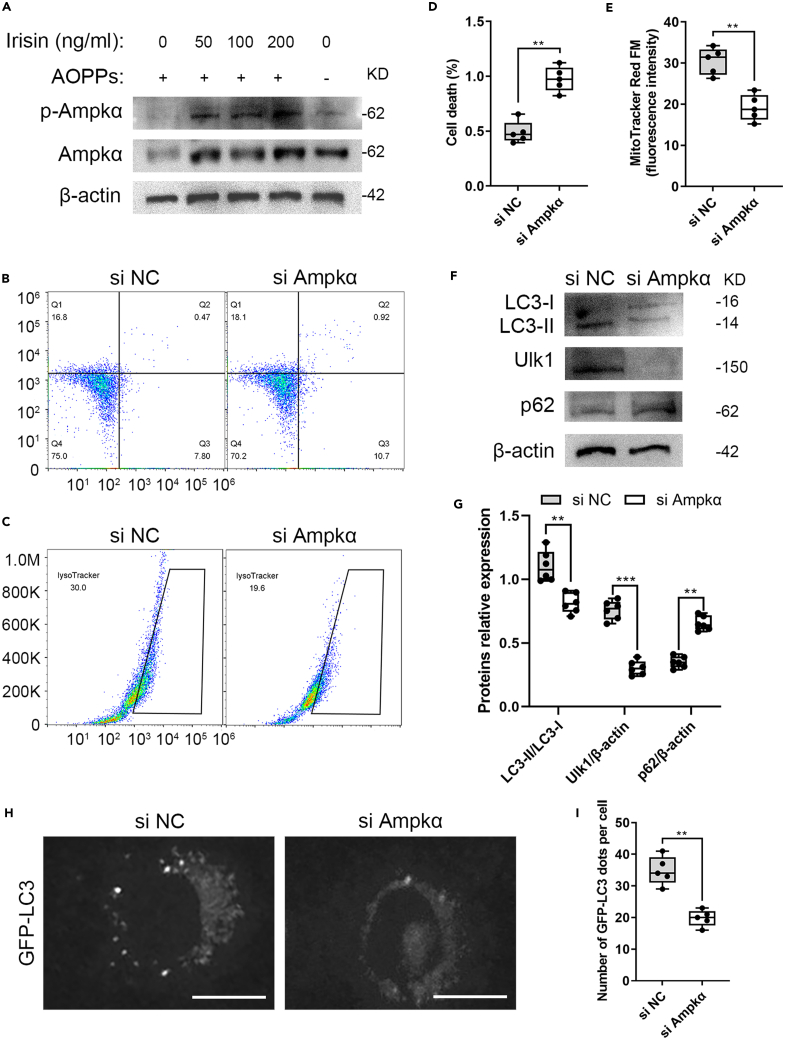
Irisin directly modulates osteocyte mitophagy through Ampk-dependent activation of Ulk1 (A) MLO-Y4 cells were treated with the indicated concentrations of irisin and 200 μg/mL AOPPs, followed by analysis of *p*-Ampkα and Ampkα protein expression using western blotting (two-tailed non-parametric Mann-Whitney test; *n* = 3 independent experiments). (B–E) MLO-Y4 cells were transfected with Ampkα siRNA and treated with 100 ng/mL irisin and 200 μg/mL AOPPs, followed by analysis of cell death and mitochondrial activity using flow cytometry (two-tailed unpaired Student’s t test; *n* = 3 independent experiments). (F and G) Western blotting confirmed that LC3-I, LC3-II, Ulk1 protein expression levels were lower, and p62 was higher in the Ampkα siRNA group compared to the control group (two-tailed non-parametric Mann-Whitney test; *n* = 3 independent experiments). (H and I) MLO-Y4 cells transiently expressing GFP-LC3 were treated with 100 ng/mL irisin and 200 μg/mL AOPPs and the average number of GFP-LC3 dots per cell was quantified between the Ampkα siRNA group and the control group (two-tailed non-parametric Mann-Whitney test; *n* = 3 independent experiments). ^∗∗^*p* < 0.01; ^∗∗∗^*p* < 0.001. Scale bar, 10 μm. Data are represented as mean ± SD.

To investigate whether irisin-induced osteocyte mitophagy is dependent on *p*-Ampkα, we evaluated the protein expression levels of *p*-Ampkα and Ampkα in the MLO-Y4 cells under oxidative stress and treatment with 50, 100, or 200 ng/mL r-irisin. We found that irisin treatment activated the protein expression of *p*-Ampkα, and the effect of irisin was dose-dependent (Figure 7A). To clarify whether Ampk signal is involved in irisin-induced phenotype switch in the osteocytes, siRNA targeting Ampkα was applied to disable Ampk activation. Flow cytometry analysis demonstrated that the Ampkα siRNA MLO-Y4 cells under oxidative stress with 100 ng/mL r-irisin significantly increased the percentage of cell death and decreased the mitochondrial activity compared to the control group (Figures 7B–7E). Western blotting analysis showed that Ampkα siRNA dramatically disable irisin-induced osteocyte mitophagy, as indicated in the lower expression of LC3-II and Ulk1, and higher expression of p62 (Figures 7F and 7G). Importantly, the MLO-Y4 cells expressed little protein level of Ulk1 because of Ampkα knockdown. Furthermore, the number of GFP-LC3 dots was significantly decreased in the Ampkα siRNA group compared to control group (Figures 7H and 7I). Taken together, the aforementioned findings indicated that increased irisin can directly modulate osteocyte mitophagy through Ampk activation and demonstrated the importance of Ampk-Ulk1 signaling in bone remolding for the senile osteoporosis.

## Discussion

Osteoporosis is the consequence of a disturbance in the existing balance between osteoblastic bone formation and osteoclastic bone resorption.^50^ Consequently, osteoporosis therapies fall into two classes, anti-resorptive drugs, which inhibit bone resorption, and anabolic drugs, which stimulate bone formation. Osteoporosis is also defined by a loss of bone strength and a reduction of bone mass with a deterioration of bone quality, leading to an increased fracture risk.^13^ The osteocyte is now regarded as being at the center of bone remodeling by coordinating both osteoblast activity and osteoclast resorption, but also as the initiator of the bone remodeling processes by locally sensing bone matrix deformation.^13^ Therefore, molecular pathways that control osteocyte functions might become potential therapeutic targets for the treatment of osteoporosis. Exercise has a positive impact in the reduction of osteocyte apoptosis.^1^^,^^2^ Irisin, being regulated by exercise, boosts the bone mass and higher irisin levels are associated with a lower rate of age-related osteoporosis in older adult patients.^3^^,^^4^^,^^5^^,^^6^^,^^7^^,^^51^^,^^52^ However, the data *in vivo* and *in vitro* experiments approving the therapeutic role of irisin in senile osteoporosis are still unknown. In this study, our observation exhibited that irisin can reduce body weight, enhance physical activity, and inhibit bone mass loss and bone microarchitecture alteration in senile osteoporosis mouse model. Especially, irisin at the same time promotes osteoclastic bone resorption, osteoblastic bone formation, and induces the protein expression of sclerostin, which upregulates the bone turnover in favor of bone formation.

With respect to the effects of irisin on bone remolding, all bone cells in the remodeling unit may be involved. For instance, low dose irisin injections, given intermittently, were shown to improve cortical BMD in mice through enhancing osteoblast differentiation.^53^ These effects were in line with other *in vitro* study, which demonstrating that irisin was able to increase the differentiation of bone marrow stromal cells into mature osteoblasts.^6^^,^^51^ In addition, recent study has demonstrated that osteocytes are direct targets of irisin, acting through the integrin αV family.^8^ Osteocytes use both mechanical and chemical sensation to maintain bone homeostasis by controlling osteoblast activity through Wnt signaling pathway and osteoclast resorption through secreting RANKL.^9^ Based on the aforementioned findings and the previous studies, we thought that the balance between bone resorption and bone formation regulated by the osteocyte was restored under the treatment of irisin. Furthermore, data presented in this study support the concept that irisin preserves osteocyte viability through inducing osteocyte autophagy/mitophagy, which plays a critical role for the maintenance of bone homeostasis,^17^^,^^23^^,^^24^^,^^25^ thereby regulating bone remolding in the senile osteoporosis.

**Figure 8:**
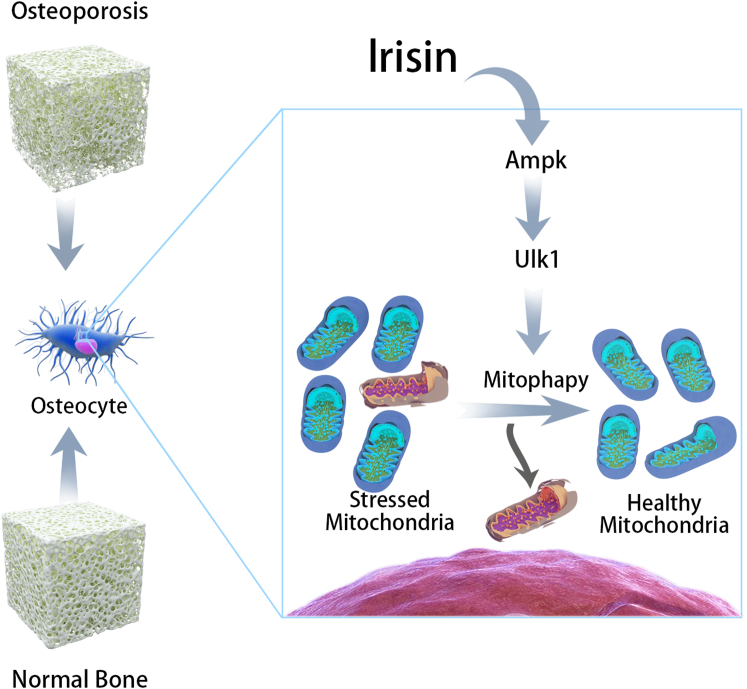
Schematic representation of irisin reduces age-related bone loss by inducing osteocyte mitophagy through Ampk-dependent activation of Ulk1

Ampk is a serine/threonine kinase and a prominent metabolic sensor in cells. At the cellular level, energy stress leads to the activation of Ampk, which inhibits energy-consuming anabolic pathways and triggers energy-promoting catabolic pathways.^54^^,^^55^ Ampk activation, during energy stress, prolongs cell survival by redox regulation.^56^ There are many evidences to support a key role for Ampk in mitophagy induction in response to various cellular stresses, including oxidative stress and exercise.^43^^,^^44^^,^^45^ Additionally, Ampk activation increases bone formation while deletion leads to decreased bone mass.^41^^,^^42^ Irisin is an exercise-induced hormone secreted by skeletal muscle that drives brown fat-like conversion of white adipose tissue, which improves systemic metabolism by increasing energy expenditure.^3^ Because of these properties, Ampk activation appears to be a potential therapeutic target for irisin in the senile osteoporosis.^46^ As might be expected, irisin treatment increased phosphorylation of Ampk and of Ulk1 in the senile osteoporosis mouse model and in the MLO-Y4 cells. Laker et al.^23^ revealed that acute exercise induces mitophagy through Ampk-dependent activation of Ulk1 in skeletal muscle. In addition, deletion of the *Ulk1* gene in skeletal muscle inhibits mitophagy without affecting lysosome formation in response to exercise. In this study, we revealed that knockdown of Ampkα dramatically restrict irisin-induced osteocyte mitophagy and eliminate the protein expression of Ulk1, a downstream regulator of mitophagy. Meantime, we also found that Ampk does not appear to be responsible for phosphorylation of Acc since we observed significant increases in phosphorylation level of Ulk1 after irisin treatment in the senile osteoporosis mouse and the MLO-Y4 cells. This suggested that Ampk phosphorylation of Ulk1 is required for targeting of osteocyte mitophagy (Figure 8), which is in line with previous reports.^23^^,^^57^

Kim et al.^8^ demonstrated that identification of irisin receptors as integrins in osteocytes and thermogenic fat suggests that αV family integrin complexes are likely to be the major irisin receptors. However, the results of previous studies on irisin were based on the ovariectomy mouse model or young mice.^6^^,^^8^ The effect of irisin on the senile osteoporosis and the possible mechanisms are not entirely known. Furthermore, there are increasing evidence that Ampk regulates integrin-dependent processes, such as cell adhesion, migration, and matrix formation via several mechanisms.^58^^,^^59^ Furthermore, Ampk may also control integrin traffic. Given that the original link between Ampk and integrin activity was observed in cancer cells,^60^^,^^61^ it is likely that this Ampk-dependent mechanism, established in fibroblasts, is also relevant in other cell types. However, the associated mechanisms of action govern the balance between Ampk and integrin remain unknown and an interesting topic for future studies.

### Limitations of the study

In summary, we demonstrate that irisin reduces age-related bone loss by inducing osteocyte mitophagy through Ampk-dependent activation of Ulk1. Understanding the precise irisin-dependent mechanisms can form the basis of developing new therapeutic strategies for prevention or treatment of the senile osteoporosis. However, this result should be further confirmed by osteocyte-specific Ampk and Ulk1 knockout mice. The role of irisin in the aging bone should be further studied in the human model.

## Resource availability

### Lead contact

Further information and requests should be directed to the lead contact, Dasheng Lin (linds@xmu.edu.cn).

### Materials availability

This study did not generate new unique reagents.

### Data and code availability

• Data reported in this paper will be shared by the lead contact upon request.
• This paper does not report original code.
• Any additional information required to reanalyze the data reported in this paper is available from the lead contact upon request.

## Acknowledgments

This study was supported by the 10.13039/501100001809National Natural Science Foundation of China (grant no. 82172477 and 81600696), the 10.13039/501100003392Natural Science Foundation of Fujian Province of China (grant no. 2023J011844, 2023J011839, 2022J011482, and 2019J01144), Fujian Medical University Union Hospital Talent Research Launch Project (grant no. 2024XH001) and the 909th Hospital Independent research project (22MS005).

## Author contributions

H.L. and W.X. performed and analyzed this research and wrote the manuscript; W.Y. performed cell culture and transfection; D.L. performed ELISA and human plasma specimens’ analyses; W.X. carried out Micro-CT and IHC analyses; J.C. performed western blotting and qPCR; P.A., M.Z., E.F., and D.D. approved manuscript; L.D. conceived the study, designed, and analyzed experiments and wrote the manuscript.

## Declaration of interests

The authors declare no competing interests.

## STAR★Methods

### Key resources table

**Table:** 

REAGENT or RESOURCE	SOURCE	IDENTIFIER
**Antibodies**		

LC3	Abcam	ab48394
Ulk1	Abcam	ab203207
p62	Abcam	ab109012
β-actin	Abcam	ab8226
*p*-Ampkα	CST	2535
Ampkα	CST	5831
*p*-Acc	CST	11818
Acc	CST	3676
Osteocalcin	ProteinTech	23418-1-AP
Sclerostin	Abcam	ab63097
horseradish peroxidase-conjugated goat anti-rabbit IgG	Thermo Fischer Scientific	31466

**Biological samples**		

α-MEM	ThermoFisher Scientific	12571–063
Fetal bovine serum	ThermoFisher Scientific	16140–071
Calf serum	ThermoFisher Scientific	A3520502

**Chemicals, peptides, and recombinant proteins**		

r-irisin	Adipogen	AG-40B-0103
Lipofectamine 2000	ThermoFisher Scientific	11668019
Protease Inhibitor Cocktail	Sigma-Aldrich	P8340
PhosSTOP Phosphatase Inhibitor Cocktail	Sigma-Aldrich	PHOSS-RO

**Deposited data**		

N/A	N/A	N/A

**Experimental models: Cell lines**		

MLO-Y4	iCell Bioscience	iCell-m037
Murine long bone-derived osteocyte-like cell line	This paper	N/A

**Oligonucleotides**		

Ampk siRNA: GCCUCACCCUGAAAGAGUATT	This paper	N/A

**Software and algorithms**		

Micro-CT	Bruker-microCT	SkyScan 1176
Spectrophotometer	Tecan Infinite	F200/M200
fluorescent temperature cycler	Roche	LC480

**Other**		

Human Irisin ELISA Kit	R&D Systems,	DY9420-05
Human AMPK ELISA Kit	Abcam	ab181422
CTX-I ELISA Kit	Elabscience	E-EL-M0372c
PINP ELISA Kit	Elabscience	E-EL-M0233c
TRAP Kit	Sigma-Aldrich	387A
DAB detection kit	CST	8059
type I collagen-coated plates	Sigma-Aldrich	08–115
MitoTracker Red FM	ThermoFisher Scientific	M22425
Osteogenic Quantification Kit	Sigma-Aldrich	ECM815
QIAGEN RNeasy Mini Kit	QIAGEN	74106
cDNA Synthesis Kit	Thermo Fischer Scientific	11904018

### Experimental model and study participant details

#### Animals

The experimental procedures were reviewed and approved by the Institutional Animal Care and Use Committees of Xiamen University. Twenty 24-month-old C57BL/6J male mice were divided into two groups randomly and kept in rooms subjected to a 12-h light-dark cycle at the constant temperature of 23°C. All mice were allowed free access to water and a standard rodent diet. Mice in experimental groups were administrated by intraperitoneal injection with recombinant irisin at doses of 10 μg kg/d for 8 weeks. While in the control group, mice were treated with normal saline in a similar manner. The general appearance, body weight and physical activity were recorded at 0 and 8 weeks for each mouse.

#### Human plasma specimens

Blood (1–2 mL) from vertebral bone marrow aspirate were collected from 12 participants (8 males and 4 females) with osteoporotic vertebral compression fracture undergoing percutaneous kyphoplasty and 10 participants with normal BMD undergoing pedicle screw fixation due to spinal fracture (Table S1). Plasma was separated by centrifugation and stored at −80°C until analysis. The protein levels of irisin and AMP activated protein kinase (AMPK) were analyzed by ELISA. This study was carried out in accordance with the guidelines of the Declaration of Helsinki. All experimental protocols were approved by our institutional review board ([2024] No. 262) and written informed consent was obtained from all study participants.

### Method details

#### Mouse specimens processing

After anesthesia with diethyl ether, the blood from the abdominal aorta of each group was collected and centrifuged to separate the serum. The levels of C-terminal telopeptide of type I collagen (CTX-I) and N-terminal propeptide of type I procollagen (PINP) were analyzed using ELISA kits according to the manufacturer’s instructions. Mouse lumbar vertebras and lower extremity from the treatment and the control group were obtained after euthanasia. The lumbar vertebras, right femurs and tibias were fixed in 4% paraformaldehyde overnight at 4°C, and scanned by Micro-CT. And then following fixation, the lumbar vertebras, right femurs and tibias were decalcified in 10% ethylene diamine tetraacetic acid (EDTA)/phosphate buffered saline (PBS) pH 8.0 for 14 days, and embedded into paraffin, and sectioned with 6 *μ*m for TRAP staining and immunohistochemistry (IHC) staining. The left femurs and tibias were frozen in liquid nitrogen, and the left femurs were used for western blotting analysis and the left tibias for quantitative PCR (qPCR).

#### Micro-CT

For Micro-CT, high-resolution Micro-CT scans were performed on the L4 lumbar vertebras, right femurs and tibias. The scanning conditions were kept identical for all the tests (resolution: 18 mm; source voltage, 65 kV; source current, 385 μA; rotation step, 0.7°). In the femur，the region of interest (ROI) of cortical bone was analyzed in 100 transverse μCT slices in the middle femur (length = 500 μm) and cancellous bone was analyzed beginning 200 μm superior to the distal growth plate and extending proximally 1000 μm in the distal femur. In the tibia, the ROI of cortical bone were scanned at the mid diaphysis (length = 500 μm) and trabecular bon were scanned starting 200 μm distal to the growth plate and extending for 1000 μm. The trabecular bone region was identified by semi-manually contouring the trabecular bone in the ROI with the help of an auto-thresholding software algorithm. Bone volume fraction (BV/TV), trabecular thickness (Tb.Th), trabecular number (Tb.N), trabecular separation (Tb.Sp) and cortical thickness (Ct.Th, mm) were assessed. BMD of the ROI of trabecular bone region was also assessed. We achieved a series of cross-sectional gray-valued images, which were used to construct three-dimensional images of bone microstructures.^17^^,^^28^

#### Histology, immunohistology and histomorphometry

For TRAP staining, the sections were deparaffinized with xylene and rehydrated with graded concentrations of ethanol. Then, the sections were stained with TRAP and counterstained with hematoxylin according to the manufacturer’s instructions. TRAP-positive cells containing more than two nuclei were counted as osteoclasts. For IHC, the sections were incubated with 0.01 M citric acid buffer (PH 6.0) antigen retrieval for 2 min at 100°C following being washed three times with 0.1 M PBS. After using 3% H_2_O_2_ to block endogenous peroxidase activity, the sections were blocked in 10% concentrated sheep serum in PBS for 1 h. Then, the sections were incubated with primary antibodies against LC3, osteocalcin, phosphorylation of Ampk, Sclerostin, and detected using DAB detection kit. In general, all histology, immunohistology and histomorphometry experiments, unless specified otherwise in the text, were reproduced in 3 sections per sample with 10 samples per group for investigation. For quantification of the Ampk-positive area, image analysis of the bone areas stained in brown was carried out using ImageJ [Ampk-positive area/per area of interest (%)].

#### Cell culture

MLO-Y4, murine long bone-derived osteocyte-like cell line, were cultured as described.^15^ Briefly, MLO-Y4 osteocyte-like cells were seeded onto type I collagen-coated plates (0.15 mg/mL) and cultured in alpha modified essential medium (α-MEM) supplemented with 5% fetal bovine serum (FBS) and 5% calf serum (CS). After several passages using these culture conditions, osteocyte-like cells with the dendritic phenotype were enriched (Figure S4). The MLO-Y4 cell line was cloned form this osteocyte-like cell enriched population by single colony isolation. Selection was based on expression of the dendritic phenotype. Cells were cultured at 37°C and 5% CO_2_. Cell was treated with irisin or other reagents at 60% cell density.^8^

#### Advanced oxidation protein products preparation

For the AOPPs preparation, human serum albumin (30 mg/mL) was incubated with 100 mM HOCl at room temperature for 30 min. The reaction was stopped by an equimolar concentration of thiosulfate to block excess unreacted HOCl, after which extensive dialysis was carried out for 24 h at 4°C. AOPPs in human serum albumin preparations were evaluated according to the literatures.^62^^,^^63^

#### Treatment and transfection

MLO-Y4 cells were seeded in type-I collagen-coated 96 well plate (3000 cells/well) in α-MEM, 1% FBS, 1% CS, on day 0. The medium was aspirated and α-MEM, 1% FBS, 1% CS containing the indicated concentration of r-irisin (0, 50, 100, 200 ng/mL) was added to the wells. After 24 h incubation, α-MEM, 0.5% FBS, 0.5% CS containing the indicated concentration of r-irisin and 200 μg/mL AOPPs were added, and the cells were incubated for 4 h. Cell viability was measured using mitochondrial-dependent reduction of 3-(4,5-dimethyl-2-thiazolyl)-2,5-diphenyl-2-H-tetrazolium bromide (MTT). The absorbance was measured by using a spectrophotometer at 490 nm.

MLO-Y4 cells were seeded in type-I collagen-coated 96 well plate (3000 cells/well) in α-MEM, 1% FBS, 1% CS, on day 0. The medium was aspirated and α-MEM, 1% FBS, 1% CS containing 100 ng/mL r-irisin was added to the wells. After 24 h incubation, α-MEM, 0.5% FBS, 0.5% CS containing 100 ng/mL r-irisin and 200 μg/mL AOPPs were added, and the cells were incubated for 4 h. To detect dead cells, the cells were stained with Annexin V-FITC and propidium iodide in binding buffer at 4°C. Samples were detected with Attune NxT (ThermoFisher Scientific, USA), and the results were analyzed using FlowJo software (TreeStar, USA). To detect the mitochondrial activity of the MLO-Y4 cells, the cells were stained with MitoTracker Red FM according to the manufacturer’s protocol, and detected with Attune NxT (ThermoFisher Scientific, USA). The results were analyzed using FlowJo software (TreeStar, USA). Quantitative histomorphometry was carried out via an automated quantitative image analysis according to algorithms from literature.^64^ In brief, using ImageJ (National Institutes of Health, USA), the following algorithm was applied, 1) area of interest was manually designated using the “drawing/selectionˮ tool; 2) “set measurementsˮ for area, integrated density and mean gray value was selected from the analyze menu; 3) lastly, the corrected total cryosections fluorescence (CTCF) representing MitoTracker Red FM were exported and calculated in Excel (Microsoft) as follows CTCF = media of integrated density – (media of area of selected area × mean fluorescence). To quantitative analysis of tubular and fragmented mitochondria, MLO-Y4 cells were grown on coverslips and then transfected with pDsRed2-Mito plasmid (MiaoLingPlasmid, P0142), which leads to the expression of a MitoRed mitochondria-targeted fluorescent protein, to label the organelles. The transfected cells were fixed in 4% paraformaldehyde for 10 min at room temperature and analyzed by fluorescence microscopy. Mitochondria was morphology in individual cell was evaluated as described.^65^

To investigate osteocyte mitophagy, MLO-Y4 cells seeded in 12 well-plates coated with type-I collagen reaching 60% confluence were transfected with mRFP-GFP-LC3 (LC3, light chain 3; MiaoLingPlasmid, P4838) using Lipofectamine 2000 Transfection Reagent for 24 h. After designated treatments, cells were fixed with 4% paraformaldehyde in PBS. All the cellular images were obtained inverted confocal microscope. For quantification of autophagic cells, GFP-LC3 and mRFP-LC3 punctated dots were determined from triplicates by counting a total of more than 30 cells.^66^^,^^67^^,^^68^

#### Gene interference with Ampk siRNA

To explore gene interference with Ampk siRNA, siRNA targeting Ampk (sense: 5′-GCCUCACCCUGAAAGAGUATT-3’; anti-sense: 5′-UACUCUUUCAGGGUGAGGCTT-3′) or control siRNA (sense: 5′-UUCUCCGAACGUGUCACGUTT-3’; anti-sense: 5′-ACGUGACACGUUCGGAGAATT-3′) were synthesized by GenePharma (Shanghai, China). Transfection was performed by using GP-transfect-Mate (GenePharma, 180403) according to the manufacturer’s protocol. Briefly, MLO-Y4 cells were seeded in 12 well-plates coated with type-I collagen reaching 60% confluence were transfected with Ampk siRNA or scramble siRNA using GP-transfect-Mate. Knockdown efficiency was confirmed by immunoblotting test (Figure S5). After 24 h incubation, α-MEM, 0.5% FBS, 0.5% CS containing 100 ng/mL r-irisin and 200 μg/mL AOPPs were added, and the cells were incubated for 4 h. Dead cells, mitochondrial activity, mitophagy and western blotting analysis were performed to determine the effect of knockdown Ampkα.

#### Osteogenic and adipogenic differentiation

For osteogenic differentiation, 8 × 10^3^ cells/cm^2^ human bone marrow mesenchymal stem cells (hBMSCs) were seeded in 6 well-plates. When the cell density reached approximately 70% confluence, growth medium was substituted with osteogenic medium consisting of α-MEM supplemented with 10% FBS, 10 mM β-glycerophosphate, 50 μM L-ascorbic acid 2-phosphate, and 100 nM dexamethasone (all from Sigma-Aldrich, Germany) for 21 days. The extent of osteogenic differentiation was determined by Alizarin Red S and quantification using Osteogenic Quantification Kit, as recommended by the manufacturer. For adipogenic differentiation, 8 × 10^3^ cells/cm^2^ hBMSCs were seeded in 6 well-plates, and were cultivated in an induction media for 5 days (α-MEM with 10% FBS, 1 μM dexamethasone, 200 μM indomethacin, 0.01 mg/mL insulin, and 500 μM 3-isobutyl-1-methylxanthine; all from Sigma-Aldrich, Germany) followed by 2 days in preservation media (α-MEM supplemented with 10% FBS, 0.01 mg/mL insulin). The process was repeated for 21 days. The adipogenic differentiation was estimated by BODIPY 493/503 staining of neutral lipid droplets (Thermo Fisher Scientific, Waltham, MA, USA) as described.^69^

#### Western blotting analysis

Fresh left femurs and MLO-Y4 cells were frozen in liquid nitrogen and homogenized in RIPA buffer [10 mM Tris (PH 7.4), 150 mM Nacl, 0.5% NP-40, 0.1% SDS, 0.1% deoxycholate, 1 mM PMSF, 2 mM sodium fluoride, and 1 mM sodium orthovanadate] supplemented with Protease Inhibitor Cocktail and PhosSTOP Phosphatase Inhibitor Cocktail. Tissue and cell lysates containing equal amounts of proteins were separated on 10% SDS-polyacrylamide gels and transferred onto PVDF membranes, which were blocked in 5% milk in PBST for 1 h. Anti-Acc, anti-Ampkα, anti-LC3, anti-*p*-Acc, anti-*p*-Ampk, anti-p62, anti-Ulk1 antibodies were added. After overnight incubation with the primary antibodies at 4°C, membranes were washed and probed with corresponding horseradish peroxidase-conjugated goat anti-rabbit IgG, and the signal was finally visualized with an enhanced chemiluminescence system. β-actin was used as an internal control.

#### qPCR

Total RNAs from the left tibias and MLO-Y4 cells were isolated with QIAGEN RNeasy Mini Kit and used for standard qPCR. For cDNA synthesis, 1 *μ*g total RNA and AMV First-Strand cDNA Synthesis Kit were implemented. The PCR primers are shown in Table S2 qPCR was performed using a fluorescent temperature cycler. The relative gene expression was calculated as a ratio to *Gapdh/GAPDH*.

### Quantification and statistical analysis

Statistical differences between two groups were determined using two-tailed unpaired Student’s t test, or two-tailed non-parametric Mann-Whitney test. In multiple comparisons, one-way ANOVA was followed by Bonferroni post-hoc correction. Results are presented as mean ± SD. Differences were considered statistically significant at the values of ^∗^*p* < 0.05, ^∗∗^*p* < 0.01, and ^∗∗∗^*p* < 0.001.

## Supplemental information

Document S1. Figures S1–S6 and Tables S1 and S2

Data S1. Statement of human samples, related to the supplemental table 1

Data S2. Statement of animal experiments, related to the STAR Methods
