# Supplementary material for: Irisin reduces senile osteoporosis by inducing osteocyte mitophagy through Ampk activation
Source: iScience. 2024 Sep 26;27(11):111042. doi: 10.1016/j.isci.2024.111042 (PMC11570468; doi:10.1016/j.isci.2024.111042)
Supplement: Document S1. Figures S1–S6 and Tables S1 and S2 [file mmc1.pdf]

iScience, Volume *27*

Supplemental information

Irisin reduces senile osteoporosis by inducing osteocyte mitophagy through Ampk activation

Honghan Li, Deqing Luo, Wei Xie, Wenbin Ye, Jinlong Chen, Paolo Alberton, Mingzhu Zhang, Eryou Feng, Denitsa Docheva, and Dasheng Lin

Supplementary Material

Supplemental Figure 1. Irisin does not accelerate their commitment towards the osteogenic differentiation in the hBMSCs, related to Figure 2.

(A and B) Alizarin Red S staining showed osteogenic differentiation of hBMSCs in the irisin treatment group and the control group (two-tailed non-parametric Mann-Whitney test; n=3 independent experiments). (C and D) q-PCR showed gene expression profiles related to osteogenic differentiation including *RUNX2* and *SP7* in the irisin treatment group and the control group (two-tailed unpaired Student's *t* test; n=3 independent experiments).

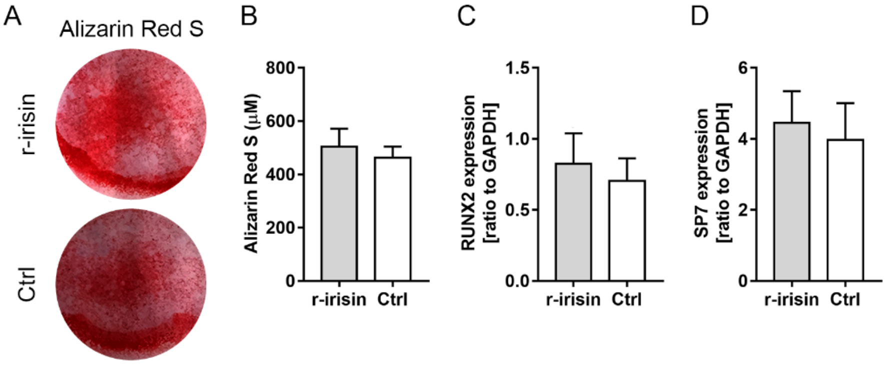

Supplemental Figure 2. Irisin does not inhibit their commitment towards the adipogenic differentiation in the hBMSCs, related to Figure 2.

(A and B) BODIPY 493/503 staining of neutral lipid droplets showed adipogenic differentiation of hBMSCs in the irisin treatment group and the control group (two-tailed non-parametric Mann-Whitney test; n=3 independent experiments). (C-E) q-PCR showed gene expression profiles related to adipogenic differentiation including *PPARγ*, *LPL* and *FABP4* in the irisin treatment group and the control group (two-tailed unpaired Student's *t* test; n=3 independent experiments). Scale bar, 200 μm.

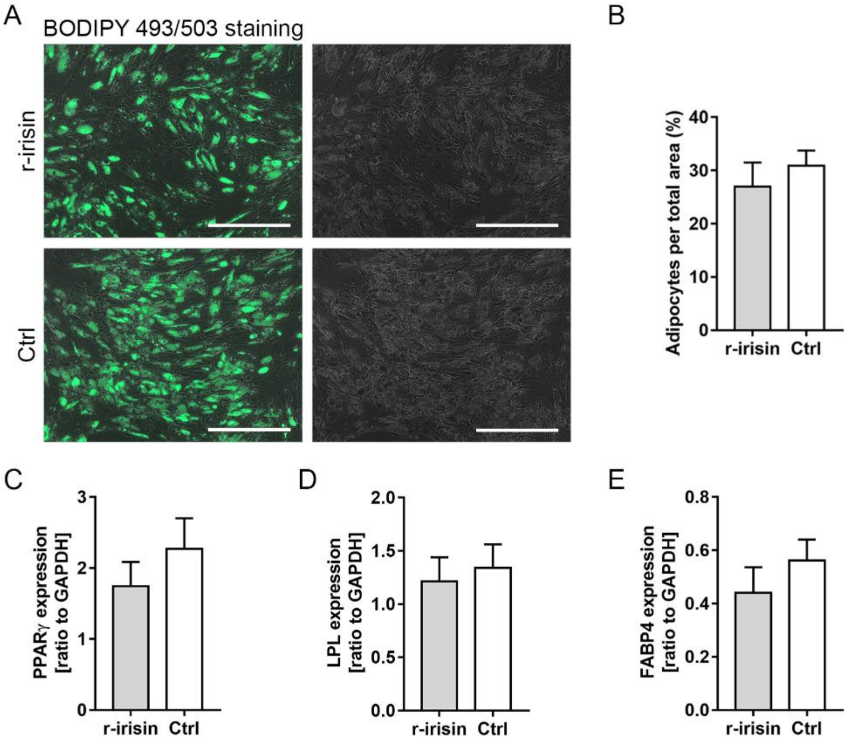

Supplemental Figure 3. Irisin induces mitochondrial activity in the MLO-Y4 cells, related to Figure 4.

(A and B) The mitochondrial activity of the MLO-Y4 cells significantly induced when compared with the control group using MitoTracker Red FM analysis (two-tailed non-parametric Mann-Whitney test; n=3 independent experiments). $^{***}p<0.001$. Scale bar, 20 μm.

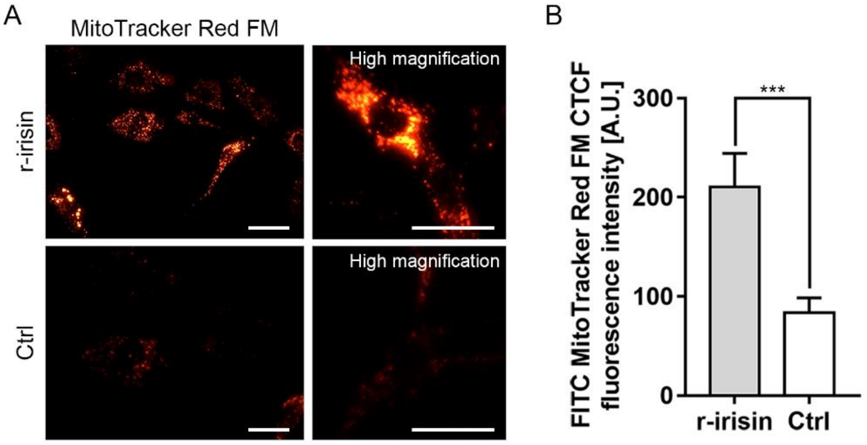

Supplemental Figure 4. Phase-contrast images of the MLO-Y4 cell line, related to Figure 4. Scale bar, 50 µm.

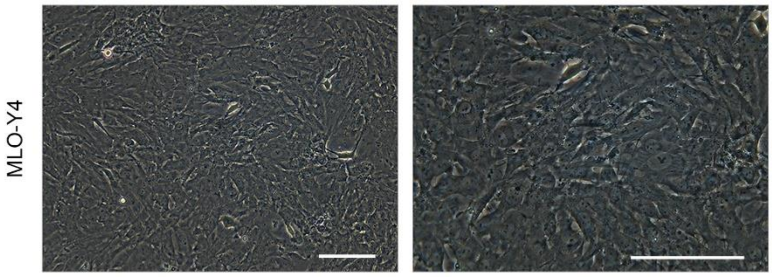

Supplemental Figure 5. The MLO-Y4 cells transfected with Ampkα siRNA were confirmed to knockdown efficiency by immunoblotting test, related to Figure 7. n=3 independent experiments.

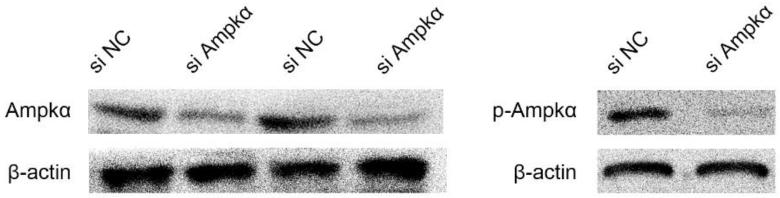

Supplemental Figure 6. The raw blots, related to Figure 3C, Figure 5C, Figure 6C, Figure 7A and Figure 7F.

Figure 3C

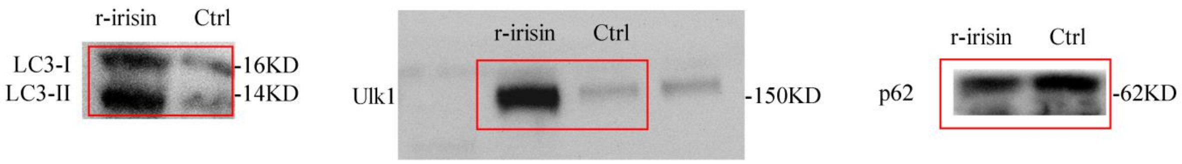

Figure 5C

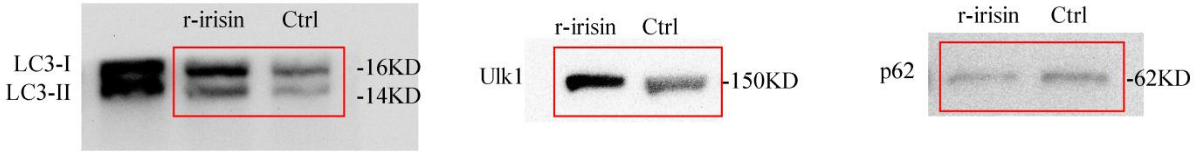

Figure 6C

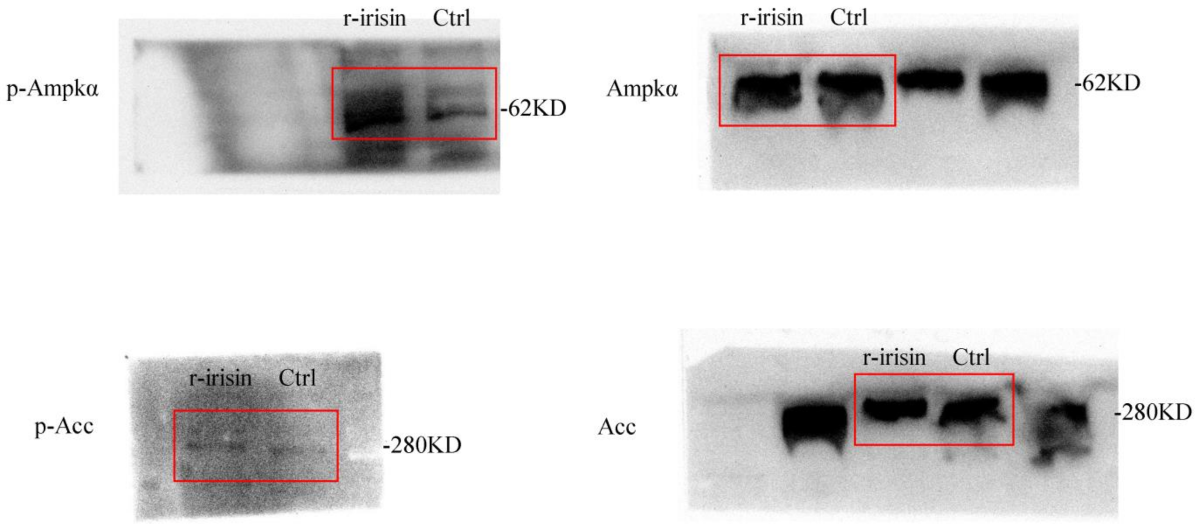

Figure 7A

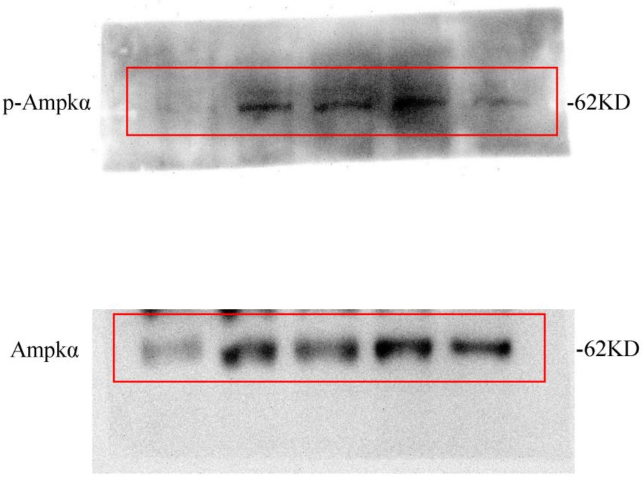

Figure 7F

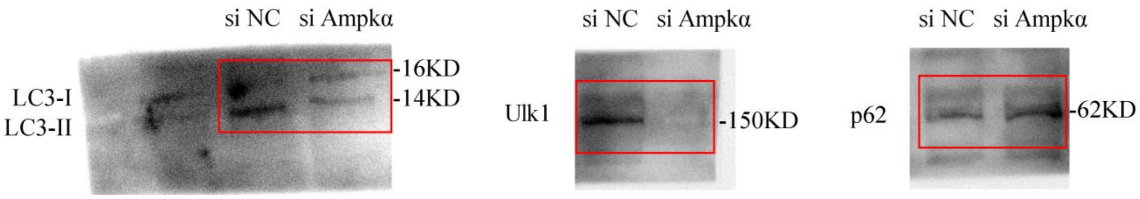

Supplemental Table 1. List of participant characteristics, related to Figure 6.

Characteristic	Normal (n=10)	Osteoporosis (n=12)	p
Mean age (range), yr	47.1 ± 1.87 (38 to 57)	69.3 ± 1.49 (61 to 78)	<0.0001
BMI (range), kg/m^2	24.3 ± 0.53 (22.4 to 27.1)	23.7 ± 0.48 (21.3 to 26.5)	0.4741
RBC count (range), ×10^{12}/l	4.31 ± 0.15 (4.35 to 4.95)	3.77 ± 0.31 (4.25 to 4.56)	0.0120
WBC count (range), ×10^9/l	5.80 ± 0.28 (4.30 to 7.10)	5.40 ± 0.22 (4.20 to 6.50)	0.2751
Hemoglobin (range), g/l	138.2 ± 4.4 (112 to 155)	117.3 ± 4.0 (98 to 144)	0.0020
L1-L4 BMD (range), g/cm^2	0.90 ± 0.02 (0.82 to 0.97)	0.52 ± 0.02 (0.42 to 0.64)	<0.0001
L1-L4 BMD (range), T value	-0.49 ± 0.08 (-0.87 to -0.12)	-3.74 ± 0.24 (-5.2 to -2.76)	<0.0001

BMD: bone mineral density; BMI: body mass index; L: lumbar vertebra; RBC: red blood cell; WBC: white blood cell

Supplemental Table 2. The primers were used in qPCR reactions.

Gene	Forward Primer (5'-3')	Reverse Primer (5'-3')
LC3	TTATAGAGCGATACAAGGGGGAG	CGCCGTCTGATTATCTTGATGAG
p62	GCTGCCCTATACCCACATCT	CGCCTTCATCCGAGAAAC
Ulk1	AAGTTCGAGTTCTCTCGCAAG	CGATGTTTTCGTGCTTTAGTTCC
Gapdh	AGGTCGGTGTGAACGGATTTG	TGTAGACCATGTAGTTGAGGTCA
FABP4	ACTGGGCCAGGAATTTGACG	CTCGTGGAAGTGACGCCTT
LPL	TCATTCCCGGAGTAGCAGAGT	GGCCACAAGTTTTGGCACC
PPARγ	TACTGTCGGTTTCAGAAATGCC	GTCAGCGGACTCTGGATTCAG

RUNX2	TCAACGATCTGAGATTTGTGGG	GGGGAGGATTTGTGAAGACGG
SP7	CCTCTGCGGGACTCAACAAC	AGCCCATTAGTGCTTGTAAAGG
GAPDH	GGAGCGAGATCCCTCCAAAAT	GGCTGTTGTCATACTTCTCATGG
