# Supplementary material for: Irisin reduces senile osteoporosis by inducing osteocyte mitophagy through Ampk activation
Source: iScience. 2024 Sep 26;27(11):111042. doi: 10.1016/j.isci.2024.111042 (PMC11570468; doi:10.1016/j.isci.2024.111042)
Supplement: Data S1. Statement of human samples, related to the supplemental table 1 [file mmc2.zip › statement of human samples.pdf]

福建医科大学附属协和医院

国家自然科学基金委员会：

经福建医科大学附属协和医院伦理委员会审定([2024]协院伦理审字第（262）号)，“运动训练上调 Irisin 调控 Ampk-Ulk1 轴激活骨细胞线粒体自噬防治老年性骨质疏松的机制”不违背伦理原则，同意实施该项研究。

福建医科大学附属协和医院伦理委员会

2024 年 3 月 1 日
