# Supplementary figures and images for: Irisin reduces senile osteoporosis by inducing osteocyte mitophagy through Ampk activation
Source: iScience. 2024 Sep 26;27(11):111042. doi: 10.1016/j.isci.2024.111042 (PMC11570468; doi:10.1016/j.isci.2024.111042)

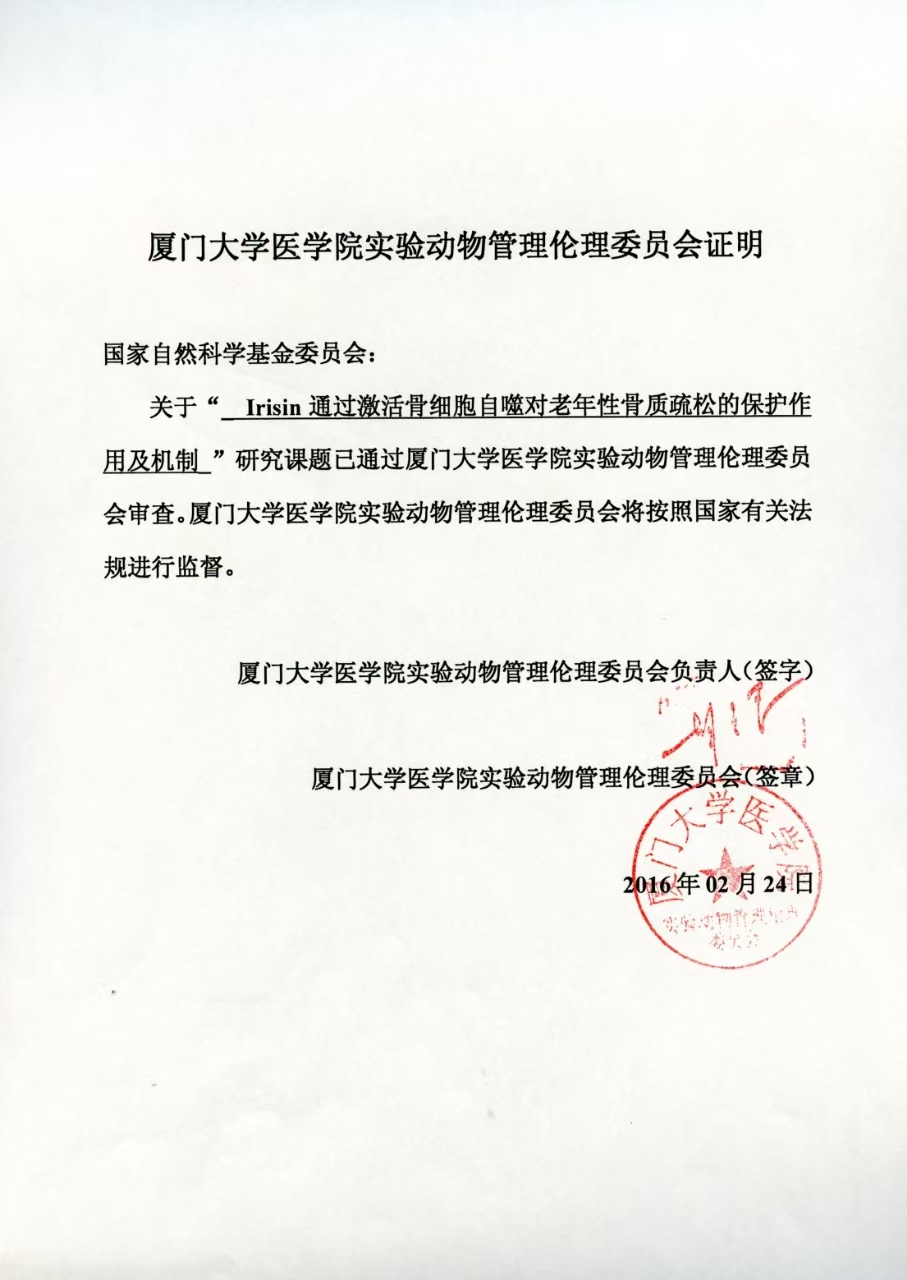

Supplement: Data S2. Statement of animal experiments, related to the STAR Methods [file mmc3.zip › statement of animal experiments.jpg]
